# Cell-level somatic mutation detection from single-cell RNA sequencing

**DOI:** 10.1093/bioinformatics/btz288

**Published:** 2019-04-26

**Authors:** Trung Nghia Vu, Ha-Nam Nguyen, Stefano Calza, Krishna R Kalari, Liewei Wang, Yudi Pawitan

**Affiliations:** 1 Department of Medical Epidemiology and Biostatistics, Karolinska Institutet, Stockholm 17177, Sweden; 2 Information Technology Institute, Vietnam National University in Hanoi, Hanoi 84024, Vietnam; 3 Department of Molecular and Translational Medicine, University of Brescia, Brescia 25125, Italy; 4 Department of Health Sciences Research, Mayo Clinic, Rochester, MN 55905, USA; 5 Department of Molecular Pharmacology & Experimental Therapeutics, Mayo Clinic, Rochester, MN 55905, USA

## Abstract

**Motivation:**

Both single-cell RNA sequencing (scRNA-seq) and DNA sequencing (scDNA-seq) have been applied for cell-level genomic profiling. For mutation profiling, the latter seems more natural. However, the task is highly challenging due to the limited input materials from only two copies of DNA molecules, while whole-genome amplification generates biases and other technical noises. ScRNA-seq starts with a higher input amount, so generally has better data quality. There exists various methods for mutation detection from DNA sequencing, it is not clear whether these methods work for scRNA-seq data.

**Results:**

Mutation detection methods developed for either bulk-cell sequencing data or scDNA-seq data do not work well for the scRNA-seq data, as they produce substantial numbers of false positives. We develop a novel and robust statistical method—called SCmut—to identify specific cells that harbor mutations discovered in bulk-cell data. Statistically SCmut controls the false positives using the 2D local false discovery rate method. We apply SCmut to several scRNA-seq datasets. In scRNA-seq breast cancer datasets SCmut identifies a number of highly confident cell-level mutations that are recurrent in many cells and consistent in different samples. In a scRNA-seq glioblastoma dataset, we discover a recurrent cell-level mutation in the PDGFRA gene that is highly correlated with a well-known in-frame deletion in the gene. To conclude, this study contributes a novel method to discover cell-level mutation information from scRNA-seq that can facilitate investigation of cell-to-cell heterogeneity.

**Availability and implementation:**

The source codes and bioinformatics pipeline of SCmut are available at https://github.com/nghiavtr/SCmut.

**Supplementary information:**

Supplementary data are available at *Bioinformatics* online.

## 1 Introduction

Cell-to-cell heterogeneity is a common feature in cancer and it has potentially important clinical consequences (Huang, 2009), but it is not possible to study this phenomena using traditional bulk-cell sequencing. Recent advances of single-cell sequencing technologies enable the study of molecular processes at cell level (Navin, 2014; Van Loo and Voet, 2014; Wang and Navin, 2015; Wen and Tang, 2016). Detection of genomic mutations using single-cell DNA sequencing (scDNA-seq) has been reported for several diseases, e.g. breast cancer (Wang *et al.*, 2014) and renal carcinoma (Xu *et al.*, 2012). However, with very low-input materials coming only from two copies of DNA molecules (Navin, 2014; Van Loo and Voet, 2014), scDNA-seq suffers many problems such as technical errors, amplification bias, low physical coverage, chimeric DNA, non-uniform coverage, allelic drop-out (ADO) event, etc (Van Loo and Voet, 2014; Wang and Navin, 2015). General analysis tools for detecting single-nucleotide variants (SNVs) from scDNA-seq data that address some of these issues have appeared recently, e.g. Monovar (Zafar *et al.*, 2016).

Single-cell RNA sequencing (scRNA-seq) has also a considerable development in recent years. Even though a mammalian cell contains a very low amount of RNAs (Wang and Navin, 2015), the number of copies of RNAs in a cell is still much greater than that of DNAs. ScRNA-seq has been widely used in investigating gene expression of cells. The information of SNV and allele-specific expression (ASE) of single cell from scRNA-seq have also been investigated recently. For example, in Kim *et al.* (2015a), the authors predict that only 17.8% stochastic ASE patterns contribute to biological noise. Similarly, Borel *et al.* (2015) report that 76.4% of heterozygous SNVs display stochastic monoallelic expression in single cells. Recently, Kim *et al.* (2015b) study the heterogeneous expression of SNVs in a study of patient-derived xenograft cells of lung adenocarcinoma.

Bulk-cell RNA sequencing (bcRNA-seq) from a population of cells has been used to detect genomic variants in many studies (Goya *et al.*, 2010; Tang *et al.*, 2014). For instance, in recent study, Piskol *et al.* (2013) report that over 70% of all expressed coding variants are identified from RNA-seq, and whole exome sequencing (WES) and RNA-seq have comparable numbers of identified exonic variants. So it is natural to investigate genomic variants from the scRNA-seq data. For example, Chen *et al.* (2016) investigate the single-cell single-nucleotide polymorphisms (SNPs) based on scRNA-seq in colon cancer. However, up to now, to our best knowledge, there are no methods specifically designed to detect cell-level somatic mutations from scRNA-seq.

In this study, we show that mutation detection methods that are developed for either bulk-cell or scDNA-seq data do not work well for the scRNA-seq data, as they produce too many false positives. We propose a novel statistical method—called SCmut—to identify cells that harbor mutations discovered in bulk-cell data. In brief, the method first collects somatic mutations from bulk-cell DNA sequencing (bcDNA-seq) of tumor and matched germline tissues. Then, combining with the collection of SNVs of single cells extracted from scRNA-seq, SCmut statistically detects the somatic mutations at cell level using the two-dimensional local false discovery rate (2D local fdr) method. We apply the method to several scRNA-seq datasets from (i) two breast cancer patients in a recent study (Chung *et al.*, 2017), (ii) two sets of cells from the breast cancer cell line MDA-MB-231, and (iii) one set of glioblastoma cells. In (i) the discovered cell-level mutations are well separated between tumor and non-tumor cells, and in (ii) the mutations are replicated in two independent datasets. In the glioblastoma dataset (iii), we discover a cell-level mutation that is highly correlated with a well-known 24 bp in-frame deletion in the PDGFRA gene. The cell-level mutation information can be used to support the characterization of cell-to-cell heterogeneity in cancer.

## 2 Materials and methods

**Fig. 1.**
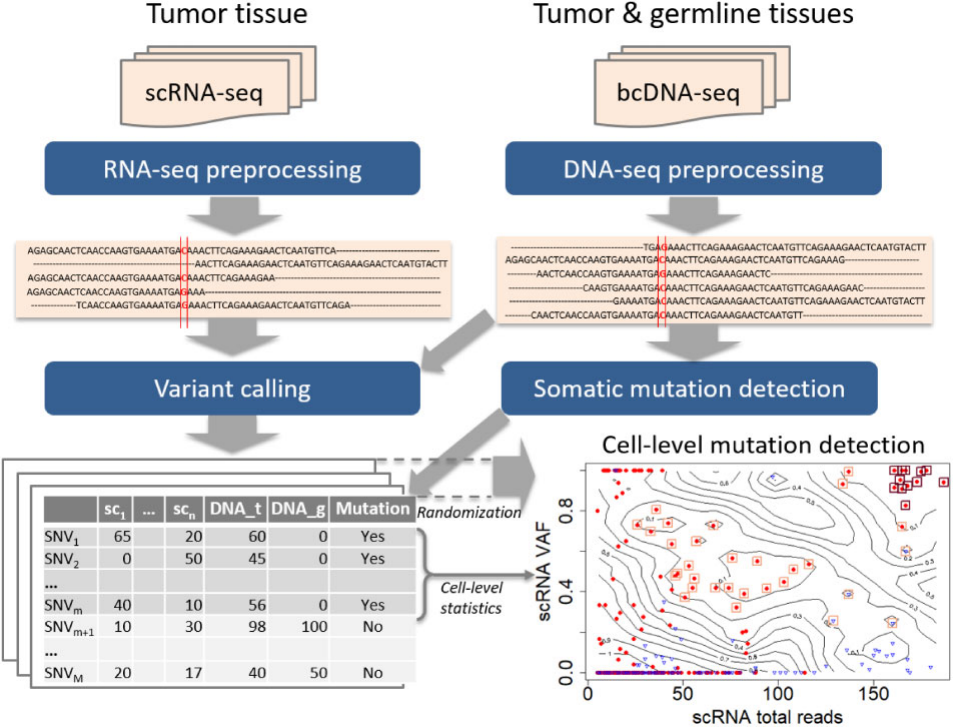
The pipeline for detecting cell-level mutation from scRNA-seq data. First, the FASTQ files of scRNA-seq and bcDNA-seq are put through preprocessing steps for alignment and clean-up to create aligned sequences in BAM files. Next the somatic mutations are detected from bcDNA-seq data, and both single-cell and bulk-cell data are put through variant calling procedures. Suppose the data contain *n* single cells and the number of obtained SNVs is *M*. Finally statistical methods, particularly the 2D local false discovery rate, are used to identify cell-level mutations. The bottom-right panel is an example of cell-level mutations discovered by SCmut from the single cells of primary tumor of patient BC03 in this study. The contour map represents the statistics from the permutation in 2D local fdr method, and each filled-circle point presents a mutation of a single cell. The red dots and blue triangles indicate the tumor cell and non-tumor cell, respectively. The significant cell-level mutations with fdr2d<0.2 and fdr2d<0.05 are marked by orange (light) and brown (dark) squares, respectively

The analysis pipeline is presented in Figure 1. First, the FASTQ files of scRNA-seq and bcDNA-seq are put through preprocessing steps for alignment, duplicate removal, recalibration, etc. to generate aligned sequences in BAM files. Next, the DNA samples of tumor and germline are used to obtain somatic mutations. Then, variant calling is implemented to all data samples of both single cell and bulk cell to get the list of SNVs. Finally, statistical methods are applied to the SNV list to discover cell-level mutations. Details of each step are presented in the following sections.

### 2.1 Data preprocessing

For DNA-seq data, which are the WES data in our examples, the FASTQ files are mapped to human hg19 annotation of Ensembl GRCh37.75 using BWA (Li and Durbin, 2009) version 0.7.10 to achieve aligned reads (BAM files). After mapping, duplicate reads are marked and removed to reduce biases from library preparation, e.g. PCR artifacts using Picard (http://broadinstitute.github.io/picard/) version 2.3.0. Realignment around indels (GATK IndelRealigner) are implemented to improve the read alignment possibly caused by mismatches. Finally, base quality scores are recalibrated (GATK BaseRecalibrator) to deal with the problems of over- or under-estimated scores caused by errors of sequencing machines. These two last steps are applied with the supports of known variant sites from Phase I of 1000 Genomes Project and dbSNP-138 (Sherry *et al.*, 2001). All the tools of GATK are implemented in GATK version 3.6.

To process RNA-seq data, the FASTQ files are also mapped to human hg19 annotation of Ensembl GRCh37.75, but using Tophat (Trapnell *et al.*, 2009) Version 2.0.12 and Bowtie2 (Langmead *et al.*, 2009) Version 2.2.3 to create BAM files. Then the follow-up processes are generally similar to the processing workflow of DNA-seq data. However, to avoid possible specific pitfalls of RNA-seq data, such as sequences overhanging into the intronic regions, after the step of removing read duplicates, an extra step (GATK Split’N’Trim) is applied. In this step, reads marked with ‘N’ symbol are eliminated and sequences overhanging regions into the intronic regions are hard-clipped.

After the preprocessing phase, the reads of RNA-seq and DNA-seq data are aligned and summarized for downstream analysis.

### 2.2 Somatic mutation detection from bcDNA-seq and variant calling

From the bcDNA-seq of tumor tissue and matched germline, the somatic mutations can be discovered by any somatic mutation detection methods, such as Mutect (Cibulskis *et al.*, 2013) or VarScan (Koboldt *et al.*, 2012), etc. For the breast cancer and GBM patients data, we detect somatic mutations with the support of the databases of known SNP and indels from Phase I of 1000 Genomes Project and dbSNP-138 (Sherry *et al.*, 2001).

Next, all samples of both single cells and bulk tissue are put through variant calling using SAMtools (Li *et al.*, 2009) version 1.3 followed by VarScan (Koboldt *et al.*, 2012) version 2.3.7. An SNV is retained for further analysis only if it has at least (i) five supporting reads, (ii) 1% variant frequency, and (iii) 15 average quality score, *for at least one sample*. For each valid SNV, we compute the cell-level statistics, including total reads and variant-allele frequency (VAF).

### 2.3 2D local false discovery rate

Ideally, a variant caller with its statistical method should achieve a high specificity and minimize the number of false positive mutations as possible. However, as we show in Section 3.1, when applied to scRNA-seq data, the traditional methods designed for DNA-seq data produce high false positive rates (FPRs). Therefore, we introduce a statistical approach to overcome this issue.

To get a procedure that is both efficient and has a good control of the false positives, we adapt a fdr2d procedure, originally developed for analysis of microarray data (Ploner *et al.*, 2006). Let denote the total reads by *z*_1_ and the VAF by *z*_2_, measured for each SNV from each cell. The fdr2d based on $z=(z_{1},z_{2})$ is defined as

(1) $$\text{fdr}2d(z_{1},z_{2})\equiv \pi_{0}\frac{f_{0}(z_{1},z_{2})}{f(z_{1},z_{2})},$$

where $f_{0}(z)$ is the 2d-density function of the statistic from the null variants, and *f*(*z*) the marginal density from all sites. The parameter *π*_0_ is the proportion of null variants; for simplicity, in the current application, we set $\pi_{0}=1$, which is conservative since that is its maximum value.

The fdr2d measures the relative contributions of null SNVs to the observed density at *z*, so it measures the rate of false discoveries if we declare the sites with observed value $z=(z_{1},z_{2})$ to be mutations. Thus we can control the false positives directly by limiting the estimated fdr2d. Since the non-mutations are not known, a key step of the method is to generate *z* by Monte Carlo sampling from non bc-mutation sites in the bc-DNA data. A non-parametric smoothing procedure is then used to estimate fdr2d.

Denote by ${\text{SNV}}_{1},\ldots ,{\text{SNV}}_{m}$ the bc-mutation sites from the bcDNA-seq data. The observed statistics are *z* values from these *m* SNVs across all single cells 1,…,n. Let Z be the *m *×* n* matrix of observed *z* values. For convenience, assume each cell of the matrix contains the pair of statistics (*z*_1_, *z*_2_). The data required to estimate $f_{0}(z)$ are based on *K* random samples, each of size *m*, of the null SNVs, i.e. the non bc-mutation sites. As for the bc-mutations we limit to SNVs with VAF=0 in the germline, since somatic mutations are not likely to have any variant in germline. Denote these samples as $Z_{1}^{*},\ldots ,Z_{K}^{*}$, representing samples of Z under the null hypothesis of no mutation. In all of the examples in this paper we use *K *=* *100 samples.

In principle, we could use non-parametric density smoothing to estimate *f*(*z*) from the observed *z’*s, and $f_{0}(z)$ from $Z_{1}^{*},\ldots ,Z_{p}^{*}$, then compute the fdr2d(z) by simple division. However, in practice this approach is problematic: at the edges of the distribution of *z* the ratio is noisy, and to control the noise, different amounts of smoothing are required for the two functions. Statistically it is better to estimate

$$r(z)\equiv \frac{Kf_{0}(z)}{f(z)+Kf_{0}(z)},$$

as the target parameter, and compute the fdr2d as

(2) $$\text{fdr}2d(z)=\pi_{0}\frac{r(z)}{K\{1-r(z)\}},$$

so only a single smoothing operation is needed. The 2d-estimation of *r*(*z*) involves:

1. treating all the statistics from $Z_{1}^{*},\ldots ,Z_{K}^{*}$ as ‘successes’ and the observed statistics from Z as ‘failures’, so that *r*(*z*) is the proportion of successes as a function of *z*.
2. Performing a non-parametric smoothing of the success-failure proportion as a function of *z*.

Further details of the 2D local fdr approach are given in the Supplementary Report.

After the fdr2d estimation, each observed candidate of the cell-level mutations has a corresponding fdr2d value (Pawitan *et al.*, 2005). The threshold of fdr2d<0.2 is typically used in our examples. Note that fdr2d is not a *P*-value, so it does not follow the usual reasoning for *P*-value thresholds. For example, if we report 10 significant mutations with fdr2d<0.2, then we expect only <2 false positives or >8 true positives. So, fdr2d<0.2 is a reasonable cutoff, while fdr2d<0.05 is too conservative and would lead to unnecessarily low sensitivity.

### 2.4 Datasets

#### 2.4.1 Breast cancer patient dataset

The full dataset from Chung *et al.* (2017) contains 11 breast cancer patients from different (predicted) molecular subtypes Luminal A, Luminal B, HER2-enriched and Basal-like. We select two patients BC03 (HER2-enriched) and BC07 (Basal-like) because they have scRNA-seq data of the tumor and lymph node tissues. In addition, we also collect the bulk-cell whole exome sequencing (bcWES) data of the primary tumor, lymph node and the matched blood. For the scRNA-seq, cells were processed by Fluidigm C1 system combining with SMARTer Ultra Low Kit for cell capture and library preparation. In bcRNA-seq, bulk RNAs were extracted from pooled cells or tumor tissues using RNeasy Plus Micro kit and prepared with SMARTer Ultra Low Kit. Then, the cDNAs libraries were sequenced using Nextera XT DNA Sample Prep Kit for cDNAs amplification following by HiSeq 2500 sequencing system (Illumina) with 100 bp paired-end read long. Each single cell from the scRNA-seq data contains 5.8 ± 1.4 million reads. The scRNA-seq data were downloaded from the NCBI Gene Expression Omnibus database under the accession code GSE75688. The bulk WES data were downloaded from the NCBI Sequence Read Archive under the accession code SRP067248.

After eliminating low quality data (Chung *et al.*, 2017), patients BC03 and BC07 contain, respectively, 33 and 50 cells from the primary tumor, and 53 and 52 cells from the lymph node. For bcWES data, the exomes are captured and sequenced by SureSelect XT Human All Exon V5 kit and HiSeq 2500 Illumina system using 100 bp paired-end mode. The coverage is 100× for tumors and 50× for blood samples. The authors also separated the cells into tumor and non-tumor (lymphocyte) cells. The further details of the datasets are referred to the original paper.

#### 2.4.2 Breast cancer cell-line dataset

The dataset includes a batch of 96 scRNA-seq samples from triple-negative breast cancer cell line (MDA-MB-231) (control group), and another batch of 96 scRNA-seq samples from the same cell line but treated with metformin (treated group). Single cells were captured and sequenced using a combination of Fluidigm protocol and Illumina HiSeq machine. There are on average 4.9 million read-pairs per cell, with read length 100 bp. Two cell groups were sequenced in separate batches, thus making them fully independent. After removing empty-cell wells (the negative controls), there remain 82 and 88 cells in the control group and the treated group, respectively. Since there are no available DNA-seq of the cancer cell line and matched normal germline, we obtain 99 confirmed-somatic mutations of MDA-MB-231 cell line from the COSMIC database (Forbes *et al.*, 2017) for downstream analysis (available in Supplementary Table S1 of the Supplementary Report).

#### 2.4.3 Glioblastoma dataset

This dataset contains 96 cells from a primary brain tumor of a glioblastoma multiforme (GBM) patient (patientID SF10282) from a recent study (Müller *et al.*, 2016). Libraries of single cells were captured and prepared on the Fluidigm C1 system then sequenced on HiSeq 2500 (Illumina) using paired-end 100 bp protocol. The bcWES data of the tumor and matched blood were sequenced using Illumina-HiSeq 2500 machine with 100 bp paired-end reads. Further details of the dataset are referred to the original publication.

## 3 Results

### 3.1 Challenges of mutation detection from scRNA-seq

#### 3.1.1 Concordance of VAFs between scRNA-seq and bulk-cell sequencing data

**Fig. 2.**
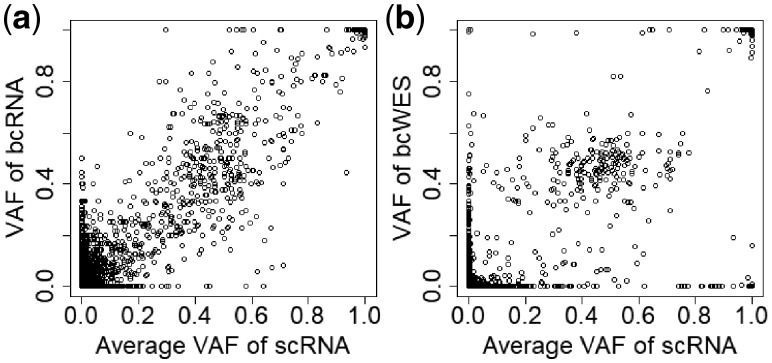
The concordance of VAFs of SNVs of scRNA-seq with bcRNA-seq (**a**) and bcWES (**b**).

To assess the quality of scRNA-seq, we first check their concordance with the more established bcDNA-seq and bcRNA-seq. Figure 2 shows the result for primary breast cancer from patient BC03, comparing the VAFs from scRNA-seq (pooled across 33 cells) against the VAFs from bcRNA-seq and bcDNA-seq (bcWES). Only common variants (present in ≥50% cells) are included. The correlation is high (*r *=* *0.89) with bcDNA-seq (Panel b), and even higher (*r *=* *0.96) with bcRNA-seq (Panel a). Thus, despite the high level of noise, scRNA-seq data can capture the underlying variant information that exists in bulk-cell data.

#### 3.1.2 High level of noise in scRNA-seq data

**Fig. 3.**
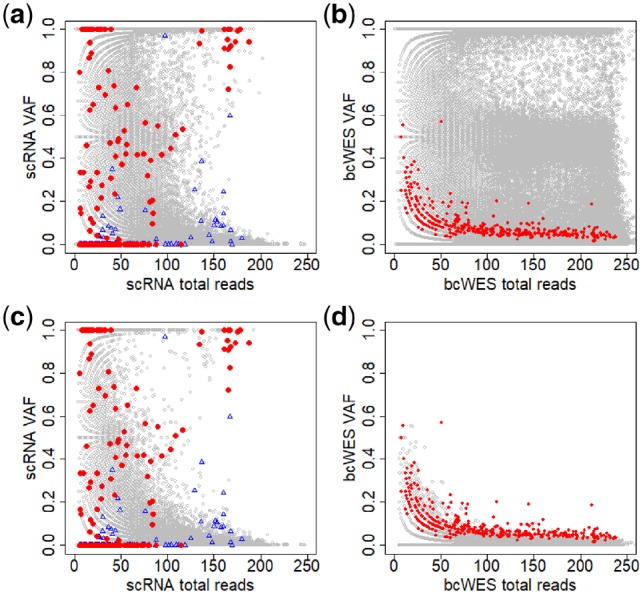
Noisy data create a great challenge in identifying mutations in single cells. (**a** and **b**) Plots of total reads versus VAFs across all SNVs for scRNA-seq and bcWES, respectively. Panels **c** and **d** are similar to Panels a and b after limiting by germline VAF=0. Gray circles are non-mutated SNVs and red dots are highlighted mutated sites detected from bcWES data. The blue triangles are highlighted mutated sites from non-tumor cells

The high proportion of stochastic monoallelic expression of SNVs is well known in scRNA-seq data (Borel *et al.*, 2015). Figure 3 displays the total reads (coverage) and VAFs of the data from the primary breast cancer of patient BC03. We first apply Mutect (Cibulskis *et al.*, 2013) to discover the mutations in the tumor from the bcWES data. The mutated sites are highlighted in the plots using red/blue colors, where the red is for the mutated sites from tumor cell and the blue is for the ones from non-tumor cell. The distributions of the non-mutated sites (gray circles) in the scRNA-seq (Panel a) and bcWES (Panel b) are similar. However, the distributions of the mutated sites (in red/blue) in the scRNA-seq and bcWES are very different.

When we include only SNVs with no germline variant (VAF=0 in germline data), the distributions of non-mutated SNVs in scRNA-seq and bcWES are now clearly different (Panels c and d). In scRNA-seq data (Panels a and c), SNVs with VAF≈1 are commonly observed across many cells. For bulk-cell data, such high VAF would be a strong evidence for mutation sites, but there is very little overlap between these SNVs and the bulk-cell mutation calls. In (Panel d), the mutation sites in bcWES are detectable as the extreme points of the distribution. But, in (Panel c), those mutation sites lie in the middle of the distribution. These features highlight the difficulty of mutation detection from scRNA-seq data alone, as the approach used for in bcWES is not likely going to work.

#### 3.1.3 Traditional methods designed for bulk-cell sequencing data

**Fig. 4.**
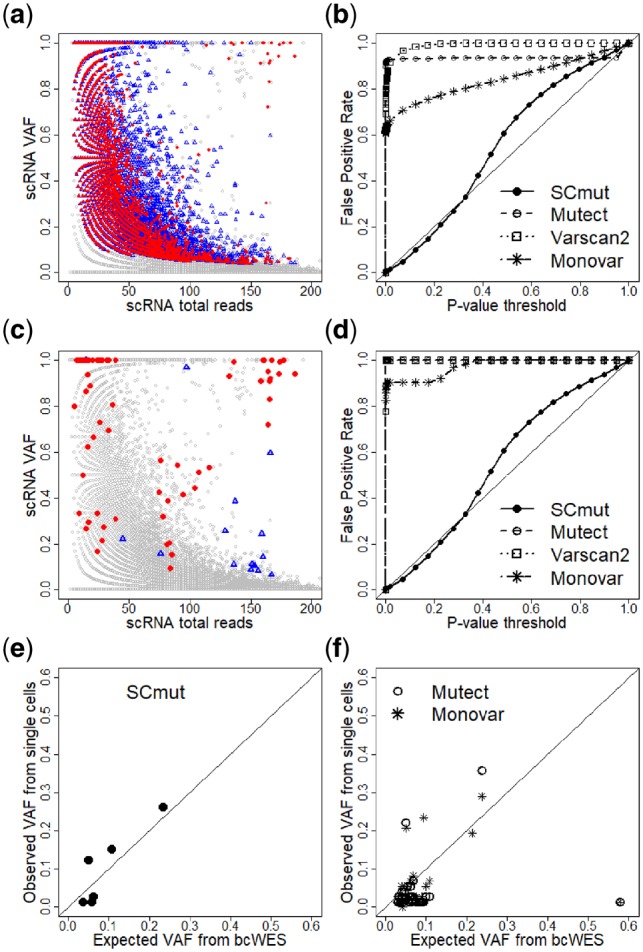
Comparison of SCmut to the other methods. (**a**) Mutations of single cells of the primary tumor of patient BC03 discovered by Mutect. Gray circles are non-mutated sites. The mutated sites are red dots for tumor cells or blue triangles for non-tumor cells. (**b**) False positive rates computed from SCmut, Mutect, Varscan2 and Monovar. Panels **c** and **d** are similar to Panels a and b, respectively, but restricted to cell-level mutations that overlap with mutations from bcWES data. Evaluation of the recovery sensitivity of SCmut and other methods (Mutect and Monovar) using expected VAF from bcWES and observed VAF estimated from single cells are displayed in **e** and **f**, respectively

To investigate further, we use Mutect (Cibulskis *et al.*, 2013), a widely used bulk-cell method to detect mutations in the single cells of the primary breast tumor of patient BC03. In particular, the scRNA-seq sample of each single cell (treated as tumor sample) and the bcWES of the blood sample (normal sample) are put through the software. We collect the detected mutations from all single cells and plot them in Figure 4a. The gray circles of the plot are non-mutated sites, and the red dots and blue triangles are the mutations from the tumor and non-tumor cells, respectively. There is a total of 25 265 cell-level mutations from 24 469 mutation sites; i.e. an average of 25 265/24 469 = 1.03 cells have mutations per mutation site, or almost all mutations are singletons (seen only in one cell). The called mutations cover the full range of VAFs and total reads above a certain value; this is an expected feature of the bulk-cell method, but clearly unsatisfactory in this case. The distributions of the mutations from tumor and non-tumor cells are highly overlapping, where many red dots share the locations with blue triangles. Moreover, the mutations from the single cells rediscover only 24 of 371 mutation calls from the bcWES. Thus, overall, the results indicate a high proportion of false positives from the mutations discovered by the standard bulk-cell method when applied to scRNA-seq data.

### 3.2 Cell-level mutations in breast cancer patient BC03

We apply SCmut to detect mutations from single cells of both primary tumor and lymph-node tissues from breast cancer patient BC03. Both primary tumor and lymph node cells have a high level of heterogeneity, where a high proportion of different immune cells (≥50%) infiltrated into the tissues (Supplementary Table S2). The types of cells (tumor or non-tumor) are identified in the original study (Chung *et al.*, 2017); we use this information to assess the specificity of the mutation calls, since we do not expect the non-tumor cells to have mutations as in tumor cells.

First, we discover 371 somatic mutations from the primary tumor using the bcWES data. Then, a total of 1 253 869 SNVs detected across the single cells and the bulk cells are used to identify cell-level mutations. The results of the fdr2d method for the single cells from primary tumor dataset are presented in the panel “Cell-level mutation detection” in Figure 1, and re-plotted in Supplementary Figure S1. The contour map of the plot represents the fdr2d estimate, and each point represents SNVs from a single cell limited to the 371 bc-mutation sites above. The red and blue points indicate the tumor cell and non-tumor cell, respectively. The significant cell-level mutations (with fdr2d<0.2) are marked by the orange squares. Most of the detected significant mutations with fdr2d<0.2 are from tumor cells, and all satisfying fdr2d<0.05 (marked by the brown squares) are tumor cells.

The results indicate the high specificity of SCmut, since few of the calls are from the non-tumor cells. Looking at the top-left part of the panel, SCmut does not call SNVs with VAF=1 as significant mutations, as would be expected in bulk-cell analysis. In single-cell data, these observations are quite common (Fig. 3a and c) due to stochastic monoallelic expression (Borel *et al.*, 2015). Thus, SCmut is robust to the intrinsic noise of the single-cell data.

**Fig. 5.**
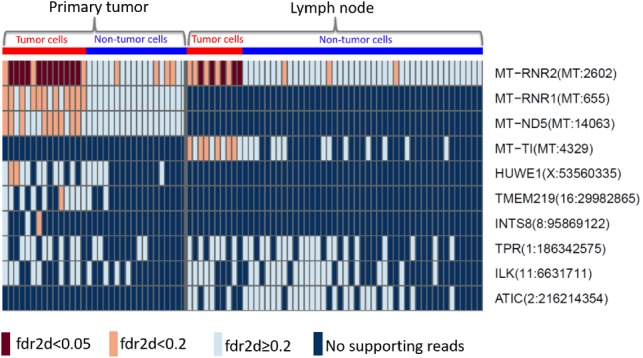
Top 10 most frequent significant mutations from the single cells of the primary tumor (left) and the lymph node (right) from patient BC03. The brown and orange boxes indicate the significant mutations with fdr2d<0.05, fdr2d<0.2, respectively. The light blue presents non-significant sites with fdr2d≥0.2. The dark blue indicates sites with no supporting reads. The red and blue at the top refer to the tumor and non-tumor groups of cells, respectively

The same pipeline is used to discover cell-level mutations in the cancer tissue from the lymph node. We compare the significant mutations (Supplementary Figure S2) with those from the primary tumor. The top 10 most frequent among the significant mutations are presented in Figure 5, the full list is supplied in Supplementary Figure S3 of the Supplementary Document. Each rectangle of the heatmap represents the information of the mutation (row) in a single cell (column). Seven-mutated genes detected from bcWES data are detected in single cells of either primary tumor or lymph node (fdr2d<0.2): MT-RNR2, MT-RNR1, MT-ND5, MT-TI, HUWE1, TMEM219 and INTS8. Among those, only the mutation of gene MT-RNR2 at position 2602 of the mitochondria chromosome is replicated in both primary tumor and lymph node.

All cell-level mutations detected with fdr2d<0.05 are from gene MT-RNR2 of the tumor cells in both tissues (Fig. 5). MT-RNR2 encodes the humanin, an anti-apoptotic peptide that can prevent the translocation of Bcl2-associated X protein (Bax) from the cytosol to mitochondria to suppress apoptosis (Guo *et al.*, 2003). It can play a role in regulating cell survival and apoptosis via interacting with insulin-like growth factor-binding protein 3 (IGFBP3) (Ikonen *et al.*, 2003). Apoptosis is an important pathway in breast cancer where the increase of apoptosis is associated with malignant tumors due to increased proliferation, high grade and negativity for estrogen receptors of breast tumors, and worse survival (Parton *et al.*, 2001). The shared mutations discovered in the primary tumor and the lymph node would identify the clone(s) that have very likely migrated from the primary tissue to the lymph node. This is of potential clinical significance, as these clones have thus already shown a local metastatic potential.

Similar analyses are applied to the single-cell datasets of the primary tumor and the lymph node tissues of patient BC07. As displayed in Supplementary Figure S4 of the Supplementary Report, there are few mutations frequently detected in both primary tumor and lymph node such as PSMD7(16:74339229), POLR2L(11:842418) and SFT2D1(6:166755986). However, none of the mutations are statistically significant with (fdr2d<0.2) in either primary tumor (Supplementary Figure S5) or lymph node (Supplementary Figure S6).

### 3.3 Comparisons with other methods

#### 3.3.1 False positive rates

We further compare SCmut to widely used bulk-cell mutation detection methods Mutect (Cibulskis *et al.*, 2013) and VarScan2 (Koboldt *et al.*, 2012), and Monovar (Zafar *et al.*, 2016), a SNV detection method designed for scDNA-seq data. We use the results from patient BC03, and first compare the FPRs. The non-tumor cells identified in the original study (Chung *et al.*, 2017) are used as negative controls, so we can estimate FPRs from the mutation calls on these cells.

First, we apply these two bulk-cell methods to discover somatic mutations from each non-tumor single cell of patient BC03 using its scRNA-seq sample (treated as tumor) and the bcWES sample of the patient’s blood (normal). Since there are no available *P*-value from results of Mutect, we infer this value from the log odds (LOD_T_ score) (Cibulskis *et al.*, 2013) of tumor. The LOD_T_ score is constructed from the likelihood ratio between the signal (true variant) and noise. Twice the log likelihood value is approximately *χ*^2^ with one degree of freedom (Pawitan, 2013), so we can compute the *P*-value for each SNV. Following the requirement of the normal sample to carry somatic mutations with high confidence (Cibulskis *et al.*, 2013), we keep only the sites with significant log odds in the normal ($\mathrm{LO}D_{N}\geq 2.3$).

Similarly, for Varscan2 we collect only somatic *P*-values from the sites with no variant in germline. For SCmut, the *P*-value is computed from the connection between the global FDR and *P*-value (Pawitan *et al.*, 2005). *P*-values for SCmut are collected from both the breast and lymph-node samples.

Monovar is run for the set of single cells from the tumor sample of patient BC03. Since Monovar was designed for scDNA-seq data, we adapt some tuning parameters to make them more appropriate for scRNA-seq data. First, ADO event is common (∼20%) in scDNA-seq (Zafar *et al.*, 2016), but not in scRNA-seq (where the drop-out event refers to the RNA transcripts, not alleles). So we set the “prior probability for ADO parameter *a* to zero”. For the “prior probability for false positive error”, which is suitable for the sequencing error rate of the RNA-seq data from Illumina HiSeq, we set *P *=* *0.003 (Schirmer *et al.*, 2016; Wall *et al.*, 2014; McElroy *et al.*, 2012). The default values are applied for the other parameters. Following the original study (Zafar *et al.*, 2016), from the set of SNVs called by Monovar, somatic mutations are filtered by the bulk-cell germline variants. Since Monovar does not provide *P*-values for SNVs of cells, we compute the *P*-values from the reported likelihoods of genotypes as follows. Following a recent study (Singer *et al.*, 2018), we first transform back the normalized and Phred-scaled likelihoods for genotypes supplied by Monovar. Each SNV site, which is assumed biallelic by Monovar, from a single-cell data *D*, has the likelihood values of three genotypes *g*, including *L*_0_ (wild-type or reference genotype), *L*_1_ (heterozygous variant) and *L*_2_ (homozygous variant), where $L_{i}\equiv P(D|g=i)$ or $L_{i}\equiv P(D|i)$ in short. Thus, the posterior probability P(g=0|D) of the wild-type can be computed as

(3) $$P(g=0|D)=\frac{P(D|0)P(0)}{\sum_{i=\{0,1,2\}}P(D|i)P(i)}.$$

The genotype prior *P*(*i*) for a single cell is taken from formula (11) of Zafar *et al*.’s for the number of cells *m *=* *1. So, we have $P(0)=\frac{1}{2}(1-\theta ),P(1)=\theta$ and $P(2)=\frac{1}{2}(1-\theta )$, where θ=0.001 is the population-level mutation rate. We consider P(g=0|D) as the local FDR of the mutation calls, which can then be converted into *P*-value, exactly as we have done for SCmut.

Figure 4b displays the FPR curves of these methods. The *y*-axis presents the observed FPR under a certain *P*-value threshold in the *x*-axis; an unbiased method should have its FPR close to the diagonal line. Both Mutect and Varscan2 have a very high FPR (>0.90) even at low *P*-value threshold (<0.1). As already described previously, this result again highlights the challenge of scRNA-seq data for the traditional mutation detection methods for bulk-cell data. Monovar has a better FPR curve, but still very high (FPR>0.7) at the same *P*-value threshold (<0.1). The FPR of SCmut tracks the target diagonal line closely, indicating that it is unbiased.

To get a fairer comparison with SCmut, we further restrict the comparison to the mutations that overlap with the somatic mutations from bcWES. Figure 4c presents the cell-level mutation status after the restriction. There remain 132, 89 and 58 single-cell mutations from Mutect, Varscan2 and Monovar, respectively. As a result, the FPRs of these methods (Fig. 4d) are similar to those without restriction (Fig. 4b). This result again indicates that the cell-level mutations are over-detected by these methods.

#### 3.3.2 Recovery sensitivity

We use the information of VAF from the bulk-cell sequencing to compare the recovery sensitivity of SCmut to the other methods. The VAF of a mutation from bcWES reflects the fraction of tumor cells with the mutation; the latter is observed in single-cell data. Therefore, we can use this correspondence for assessing the sensitivity of the methods. The mutations called by SCmut (fdr2d<0.2), Mutect and Monovar that are concordant to the calls of bcWES are collected from the primary tumor data of patient BC03. To avoid the effects of copy number variants, we collect the data of copy number variants from Supplementary Data 2 of the original study (Chung *et al.*, 2017) and exclude all mutations in regions not having two copies in the tumor sample. Figure 4e and f shows the results. For SCmut, the observed VAF estimated from the cells with the mutation calls is highly concordant (*r *=* *0.89) with the expected fraction from the bcWES. The correlation is significantly higher than that from Mutect (*r *=* *0.17) and Monovar (*r *=* *0.20). Thus, for the cell-level mutations, SCmut shows a better recovery sensitivity than Mutect and Monovar.

### 3.4 Cell-level mutations in the breast cancer cell line

**Fig. 6.**
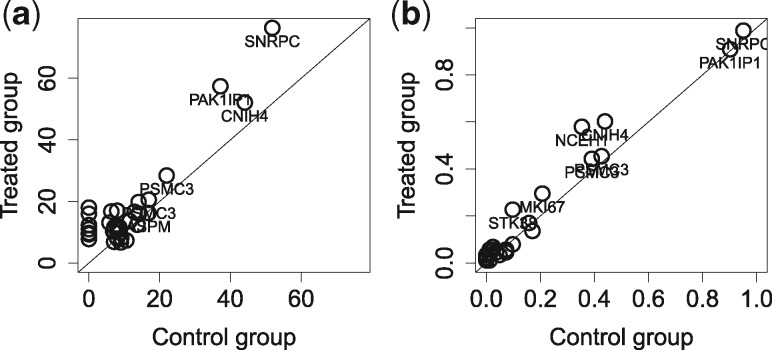
Comparison of the mutation calls of single cells between the control group and the treated group in the MDA-MB-231 dataset. (**a**) The average (across all single cells) of read coverage of the mutations is shown. (**b**) The proportions of recurrences of significant cell-level mutations with fdr2d<0.2 in the control group (*x*-axis) and the treated group (*y*-axis) are shown. Each circle in the panels presents one mutation. For convenience, only gene names of the mutations with high frequent/coverage are displayed

We apply SCmut to the breast cancer cell line (MDA-MB-231) datasets which have highly homogeneous cell populations. Results of fdr2d are presented in Supplementary Figure S8 for the control group and Supplementary Figure S9 for the treated group. A total of 99 somatic mutations (in exon region) from the COSMIC database are used as the bc-mutation sites. We observe 26 and 34 SNVs that overlap with the COSMIC sites in the control group and the treated group, respectively. All mutations in the control group are replicated in the treated group (Supplementary Fig. S9). There is a high concordance in the coverage of mutation between two groups (Fig. 6a). Moreover, some mutations with high coverage from genes CNIH4, PAK1IP1 and SNRPC can be preserved up to more than 90% of cells, indicating positive controls (Supplementary Fig. S10). We compare the recurrences of significant cell-level mutations (fdr2d<0.2) between two groups by their proportion, i.e. the proportion of cells sharing the same mutation site, in Panel b. The minor variation of the mutations to the diagonal line indicates a high correlation of the recurrent proportions between two groups (*r *=* *0.98). Thus, there are no significant effects of the metformin on the somatic mutations of MDA-MB-231 cell line. In other words, the cell-level mutations detected by SCmut are consistent between two homogeneous cell populations of the breast cancer cell line.

### 3.5 Cell-level mutations in the glioblastoma dataset

**Fig. 7.**
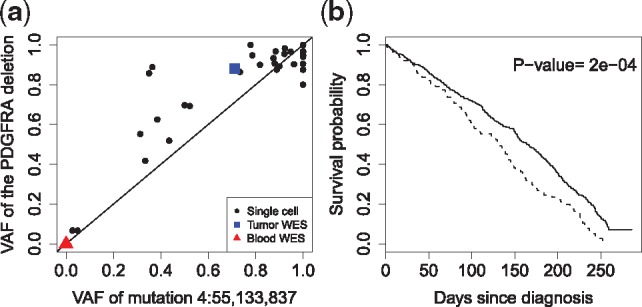
Analysis of cell-level mutations of the glioblastoma dataset. (**a**) The plot of VAFs between the point mutation chr4:55,133,837 and the in-frame 24 bp deletion in gene PDGFRA, having a high Pearson correlation of 0.91. The VAF of the deletion is the proportion of reads that support the deletion event. (**b**) The overall survival of the TCGA-GBM patients with mutations in the genes discovered by SCmut, including PDGFRA, DYNC1LI2 and CHD6 (dashed curve) versus the wild-type group (solid)

The results of SCmut to the glioblastoma dataset are given in Supplementary Figures S11 and S12 of the Supplementary Report. SCmut detects a total of 104 cell-level mutations with fdr2d<0.2. We discover one highly recurrent mutation at chr4:55,133,837, inside the PDGFRA gene, and found in 31 single cells. Intriguingly this mutation is highly correlated with a well-known 24 bp in-frame deletion in exon 7 of PDGFRA, which is also recurrent with many cells expressing PDGFRA (Müller *et al.*, 2016). The corresponding VAFs, shown in Figure 7a, have a Pearson correlation of 0.91. While we do not understand its biological significance, statistically the high correlation between these two events indicates the good sensitivity of SCmut for detecting the mutation events.

We further investigate the clinical impact of the top three recurrent-mutated genes discovered by SCmut, including PDGFRA, DYNC1LI2 and CHD6 (Supplementary Figure S13). We extract the mutation status of these genes as called by Mutect in a glioblastoma study TCGA-GBM (Brennan *et al.*, 2013) from the TCGA project (https://portal.gdc.cancer.gov/). Figure 7b shows that these mutations together are associated with poor overall survival (*P*-value = 2e−04). The results for individual genes are given in Supplementary Figure S13 of the Supplementary Report.

## 4 Discussion and conclusion

We have proposed a novel method (SCmut) to identify cell-level mutations from scRNA-seq. We present the challenges of identifying mutations from single cells, showing high levels of noise and discordances between the single-cell and bulk-cell data. Traditional mutation detection methods developed for bulk-cell sequencing data are shown to produce substantial number of false positives if applied to scRNA-seq data. We use the 2D local fdr statistic to deal with the multiple testing issues and control the false positives.

For breast cancer patient BC03, we discover one mutation from the humanin gene, an associated apoptosis gene in the mitochondrial chromosome, highly preserved in the tumor cells of both the primary tumor and lymph node. In addition, our results show that the detected cell-level mutations are well separated for tumor cells from non-tumor cells in the highly heterogeneous patient-derived cell populations, and consistent in the homogeneous cell-line populations. For the glioblastoma data example, we discover a cell-level mutation that is highly correlated with a well-known in-frame deletion, while the three top-ranking cell-level mutated genes are associated with poor patient survival.

Mutation detection from scRNA-seq data has some limitations. First, the cell-level mutations must be in the exonic regions. This is a general disadvantage of all approaches to detect mutations from RNA sequencing or WES data. Second, the procedure is highly dependent on the quality of the alignment and hence the completeness of the transcriptome annotation. Third, the stochastic monoallelic expression (Borel *et al.*, 2015) might limit the expression of the mutation sites in single cells. Finally, the detection sensitivity of a mutation is determined by the corresponding gene expression in the cell. An important mutation is statistically detectable from scRNA-seq only if it belongs to a highly expressed gene. It is challenging to assess cell-level mutations in genes with low or no expression, since the low expression could be a loss-of-function effect, but could also be due to the low coverage of scRNA-seq data, leading to false negatives. Hence, in order to detect cell-level mutation events, we recommend high-coverage scRNA-seq. Further discussion about the detection sensitivity and the coverage threshold of SCmut is presented in the Supplementary Document.

It is worth noting that SCmut focuses on detection of somatic SNVs where the sites are homozygous in normal sample but heterozygous in the tumor sample. Other types of variants such as single-nucleotide polymorphism (variants between normal samples) and homozygous SNVs (variants that are heterozygous in the normal sample but homozygous in the tumor sample) are not in the scope of this study.

To conclude, this study demonstrates that cell-level mutations can be detected from scRNA-seq data using SCmut. The identified mutations specific to cells can facilitate the characterization of the cell-to-cell heterogeneity, for instance in identifying tumor/non-tumor cells, assessing individual drug-response, profiling cell subclones, etc.

## Supplementary Material

btz288_Supplementary_Data
