# Supplementary material for: Cell-level somatic mutation detection from single-cell RNA sequencing
Source: Bioinformatics. 2019 Apr 26;35(22):4679–87. doi: 10.1093/bioinformatics/btz288 (PMC6853710; doi:10.1093/bioinformatics/btz288)
Supplement: btz288_Supplementary_Data [file btz288_supplementary_data.pdf]

Supplementary documents for "Cell-level somatic mutation detection from single-cell RNA-sequencing"

By Trung Nghia Vu, et al

Details of 2D local false discovery rate estimation

Here we describe in detail the estimation of 2D local false discovery rate (fdr2d) in the context of mutation detection from scRNA-seq data. For completeness and clarity, some parts from the main text are repeated.

Denote by $\text{SNV}_1, \ldots, \text{SNV}_m$ the bc-mutation sites from the bcDNA-seq data. The observed statistics are z values from these m SNVs across all single cells $1, \ldots, n$. Let $\mathbf{Z}$ be the $m \times n$ matrix of observed z values. For convenience, assume each cell of the matrix contains the pair of statistics (z_1, z_2). The data required to estimate $f_0(z)$ are based on K random samples, each of size m, of the null SNVs, i.e. the non bc-mutation sites. As for the bc-mutations we limit to SNVs with VAF $= 0$ in the germline, since somatic mutations are not likely to have any variant in germline. Denote these samples as $\mathbf{Z}_1^*, \ldots, \mathbf{Z}_K^*$, representing samples of $\mathbf{Z}$ under the null hypothesis of no mutation.

The procedure first estimates a 2D function

$$r(z) \equiv \frac{K f_0(z)}{f(z) + K f_0(z)},$$

and then computes the fdr2d as

$$\text{fdr2d}(z) = \pi_0 \frac{r(z)}{K\{1 - r(z)\}}, \quad (1)$$

The 2d-estimation of $r(z)$ involves:

1. treating all the statistics from $\mathbf{Z}_1^*, \ldots, \mathbf{Z}_K^*$ as 'successes' and the observed statistics from $\mathbf{Z}$ as 'failures', so that $r(z)$ is the proportion of successes as a function of z.
2. performing a nonparametric smoothing of the success-failure proportion as a function of z.

Because the full set of permuted data is large, to speed up computations, we pre-bin the data of the two dimensional grids, and the smoothing procedure to estimate the fdr2d is implemented on the grids. Since SNVs with small VAF ($< c$) are not likely genuine mutations, the corresponding fdr2d values are set to 1; in practice we use the cutoff $c = 0.2$.

From hereon the theory follows Pawitan (2001, Section 18.10). Let y_{ij} be the number of successes in the (i, j) location of the grid, and N_{ij} the corresponding total number of points that fall in the (i, j) location. By construction, y_{ij} is binomial with size N_{ij} and probability r_{ij}, the discretized version of $r(z)$. Given a smoothing parameter λ, the smoothed estimate of r_{ij} is the minimizer of the penalized log-likelihood

$$\log L(r, \lambda) = -\sum_{ij} \{y_{ij} \log r_{ij} + (N_{ij} - y_{ij}) \log(1 - r_{ij})\} + \lambda \sum_{(i,j)\sim(k,l)} (\eta_{ij} - \eta_{kl})^2$$

where $(i, j) \sim (k, l)$ means that (i, j) and (k, l) are primary neighbors in the 2D grids. The estimate is computed using the iteratively weighted least-squares (IWLS) algorithm, which

is a very stable algorithm in this case. Define the following arrays:

$$
\begin{aligned}
Y &\equiv \text{vec}(y_{ij}) \\
r &\equiv \text{vec}(r_{ij}) \\
\Sigma &\equiv \text{Diag}[N_{ij} r_{ij}(1 - r_{ij})],
\end{aligned}
$$

and R is the relationship matrix representing the primary neighbors (i.e. North, South, East and West) in the 2D grids. Let k or l be the 1D index of vector Y; the elements of $R^{-1} \equiv \text{Diag}[e_{kl}]$ are given by

$$
\begin{aligned}
e_{kk} &= \text{number of primary neighbors of } k \\
e_{kl} &= -1 \text{ if } l \text{ is a primary neighbor of } k, \text{and 0 otherwise.}
\end{aligned}
$$

Starting with an initial estimate $r^{(0)}$ needed to compute the variance matrix Σ, the IWLS updating equation is

$$r^{(1)} = (\Sigma^{-1} + \lambda R^{-1})^{-1} \Sigma^{-1} Y.$$

For speed, a fast inversion algorithm based on the Gauss-Seidel algorithm is used. At convergence, the output of the algorithm is a smooth estimate of $r(z)$, evaluated at discrete points (i, j). The fdr2d is then computed using (1), then interpolated at each observed z. The amount of smoothing as a function of λ is assessed by the effective number of degrees of freedom (df), computed using

$$\text{df} = \text{trace}\{(\Sigma^{-1} + \lambda R^{-1})^{-1} \Sigma^{-1}\}.$$

In practice, we use a relatively coarse grid on the order of $25\times$ 25 points, and λ is chosen so that df is approximately 70-80% of the number of grid points.

References

Pawitan,Y. (2001) *In All Likelihood: Statistical Modelling and Inference Using Likelihood.* Oxford University Press.

TABLES

Table S1: List of 99 confirmed-somatic mutations of the MDA-MB-231 cell-line from the COSMIC database

Position_hg19	Position_hg38	ref	alt	contig	Gene
16:2347421	16:2297420	C	G	16	ABCA3
1:94502904	1:94037348	G	A	1	ABCA4
4:175898171	4:174977020	C	A	4	ADAM29
18:56191223	18:58523991	G	A	18	ALPK2
1:145473530	1:145961557	C	T	1	ANKRD34A
1:197074117	1:197104987	T	C	1	ASPM
1:154315369	1:154342893	C	T	1	ATP8B2
6:90661568	6:89951849	G	T	6	BACH2
7:140481417	7:140781617	G	T	7	BRAF
10:124692048	10:122932532	G	T	10	C10orf88
20:31690818	20:33103012	G	C	20	C20orf186
9:139929258	9:137034806	C	A	9	C9orf139
19:42383297	19:41879227	G	A	19	CD79A
7:143018844	7:143321751	G	C	7	CLCN1
1:224553630	1:224365928	T	C	1	CNIH4
13:111134863	13:110482516	G	C	13	COL4A2
2:228145243	2:227280527	G	T	2	COL4A3
15:91185244	15:90642012	C	G	15	CRTC3
1:197611911	1:197642781	A	G	1	DENND1B
7:95709774	7:96080462	C	A	7	DYNC1I1
2:27587647	2:27364780	G	C	2	EIF2B4
3:89499345	3:89450195	G	T	3	EPHA3
19:17873592	19:17762783	G	A	19	FCHO1
10:93668048	10:91908291	C	A	10	FGFBP3
16:90108924	16:90042516	A	G	16	GAS8
1:35250656	1:34785055	G	A	1	GJB3
16:27473769	16:27462448	G	A	16	GTF3C1
4:2238074	4:2236347	G	C	4	HAUS3
4:996138	4:1002350	T	C	4	IDUA
17:42463246	17:44385878	C	G	17	ITGA2B
10:7684028	10:7642065	T	G	10	ITIH5_ENST00000397145
2:42671535	2:42444395	G	A	2	KCNG3
19:51512761	19:51009505	G	T	19	KLK9
6:63990177	6:63280272	G	T	6	LGSN
19:5699098	19:5699087	G	A	19	LONP1
1:113655185	1:113112563	A	G	1	LRIG2
12:40753186	12:40359384	T	G	12	LRRK2
12:91502008	12:91108231	C	G	12	LUM
X:30254741	X:30236624	G	A	X	MAGEB3
X:135308130	X:136225971	C	T	X	MAP7D3
10:129902653	10:128104389	T	C	10	MKI67

11:102662148	11:102791417	C	G	11	MMP1
11:59828671	11:60061198	C	A	11	MS4A3
5:80109433	5:80813614	G	A	5	MSH3
8:15967689	8:16110180	G	C	8	MSR1
8:121458742	8:120446502	G	A	8	MTBP
3:172365720	3:172647930	G	A	3	NCEH1
22:30057209	22:29661220	G	T	22	NF2
19:56321531	19:55810165	G	C	19	NLRP11
19:15300174	19:15189363	A	T	19	NOTCH3
X:30327002	X:30308885	C	A	X	NR0B1
11:64402898	11:64635426	G	T	11	NRXN2
1:114524167	1:113981545	G	A	1	OLFML3
5:180166905	5:180739905	G	T	5	OR2Y1
19:8841481	19:8731119	G	T	19	OR2Z1
11:123810829	11:123940122	G	C	11	OR4D5
11:4661717	11:4640487	C	G	11	OR51D1
6:10702647	6:10702414	G	T	6	PAK1IP1
10:55583094	10:53823334	A	T	10	PCDH15
11:117100406	11:117229690	C	G	11	PCSK7
4:55129981	4:54263814	A	T	4	PDGFRA
12:123481962	12:122997415	G	A	12	PITPNM2
22:46652694	22:46256797	G	T	22	PKDREJ
22:46652691	22:46256794	G	A	22	PKDREJ
2:160885397	2:160028886	G	C	2	PLA2R1
2:95943702	2:95277954	C	T	2	PROM2
11:47444153	11:47422602	G	T	11	PSMC3
11:47446725	11:47425174	G	C	11	PSMC3
10:62645965	10:60886207	G	T	10	RHOBTB1
6:4996598	6:4996364	C	T	6	RPP40
19:39077183	19:38586543	G	T	19	RYR1
5:54640988	5:55345160	A	T	5	SKIV2L2
2:219249912	2:218385189	T	A	2	SLC11A1
6:34730386	6:34762609	G	C	6	SNRPC
16:2814371	16:2764370	C	T	16	SRRM2
6:36463589	6:36495812	T	C	6	STK38
14:64593156	14:64126438	C	A	14	SYNE2
6:149699796	6:149378660	C	T	6	TAB2
16:89972658	16:89906250	C	G	16	TCF25
20:61488922	20:62857570	G	A	20	TCFL5
15:29997732	15:29705528	G	T	15	TJP1
9:35706092	9:35706095	A	T	9	TLN1
6:47251976	6:47284240	C	T	6	TNFRSF21
17:7577099	17:7673781	G	A	17	TP53
6:41121537	6:41153799	C	A	6	TREML1
5:14502720	5:14502611	A	G	5	TRIO
5:14330953	5:14330844	C	G	5	TRIO

6:41011373	6:41043634	C	A	6	TSPO2
20:30510808	20:31923005	G	A	20	TTLL9
3:48646621	3:48609188	G	T	3	UQCRC1
17:48918070	17:50840709	A	T	17	WFIKKN2
12:970240	12:861074	G	T	12	WNK1
2:135744526	2:134986956	T	G	2	YSK4_ENST00000375845
14:64989274	14:64522556	A	T	14	ZBTB1
4:4317443	4:4315716	G	A	4	ZBTB49
6:43325445	6:43357707	G	A	6	ZNF318
16:49670033	16:49636122	C	A	16	ZNF423
8:37556023	8:37698505	G	T	8	ZNF703
17:80789793	17:82831917	G	A	17	ZNF750

Table S2: The heterogeneity of single cells populations of the primary tumors and lymph nodes of patients BC03 and BC07: proportions of tumor cells vs non-tumor cells.

	BC03		BC07	
	**Primary tumor**	**Lymph node**	**Primary tumor**	**Lymph node**
Non-tumor	0.55	0.81	0.52	0.5
Tumor	0.45	0.19	0.48	0.5

FIGURES

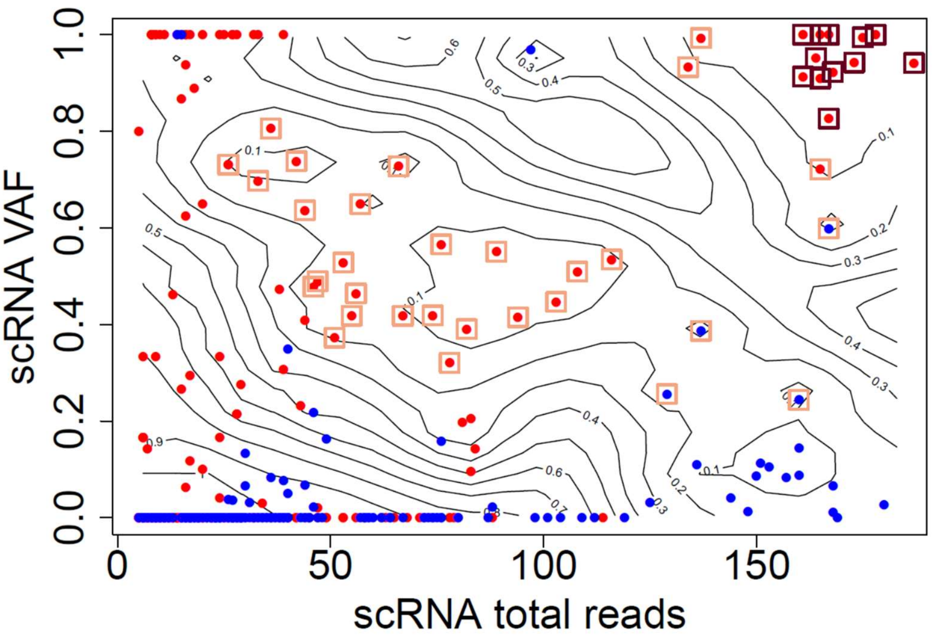

Figure S1. Results of fdr2d from the single cells of primary tumor of patient BC03. The contour map represents the statistics from the permutation in 2D local FDR method, and each filled-circle point presents a mutation of a single cell. The red and blue points indicate the tumor cell and non-tumor cell respectively. The significant cell-level mutations with fdr2d<0.2 and fdr2d<0.05 are marked by orange and brown squares, respectively.

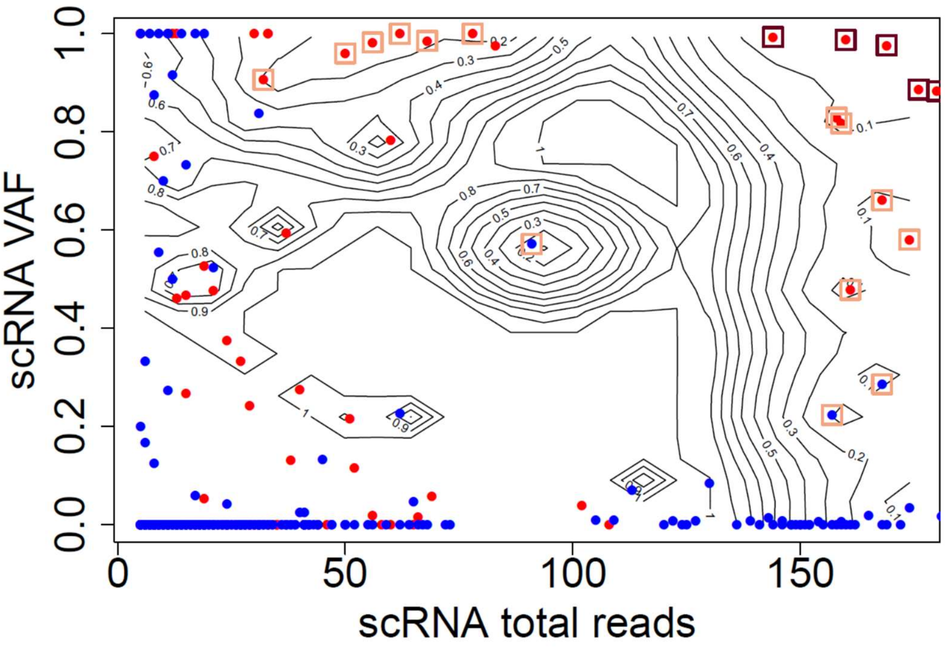

Figure S2. Results of fdr2d from the single cells of lymph node of patient BC03. The annotation is referred to Figure S1.

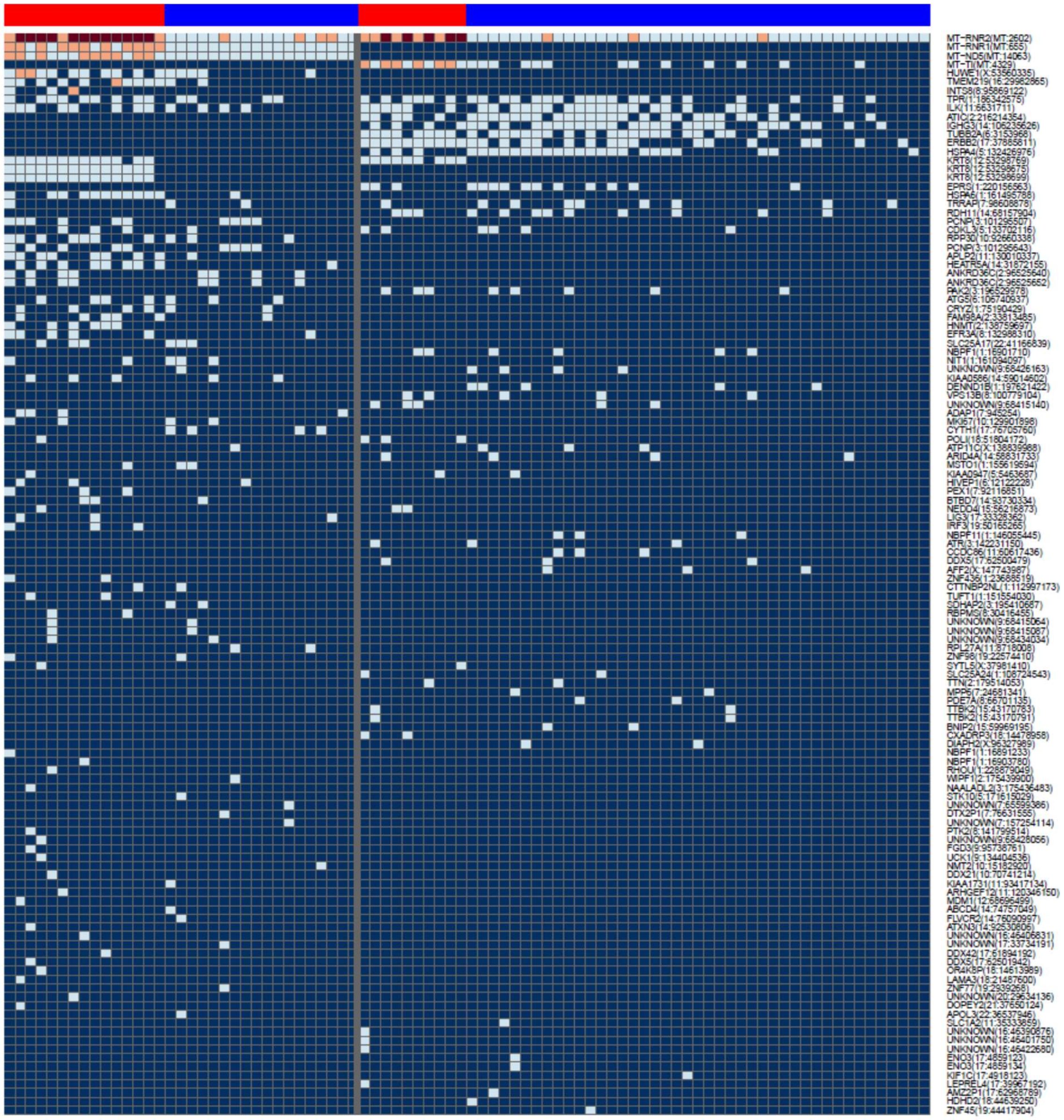

Figure S3: The full list of cell-level mutations of both primary tumor (left) and the lymph node (right) of patient BC03. The brown and orange indicate the significant mutations with fdr2d < 0.05, fdr2d < 0.2, respectively. The light blue presents the insignificant mutations fdr2d >= 0.2. The dark blue indicates sites with no supporting reads. The red and blue at the column-side-color band (top) refer the tumor and non-tumor groups of cells, respectively.

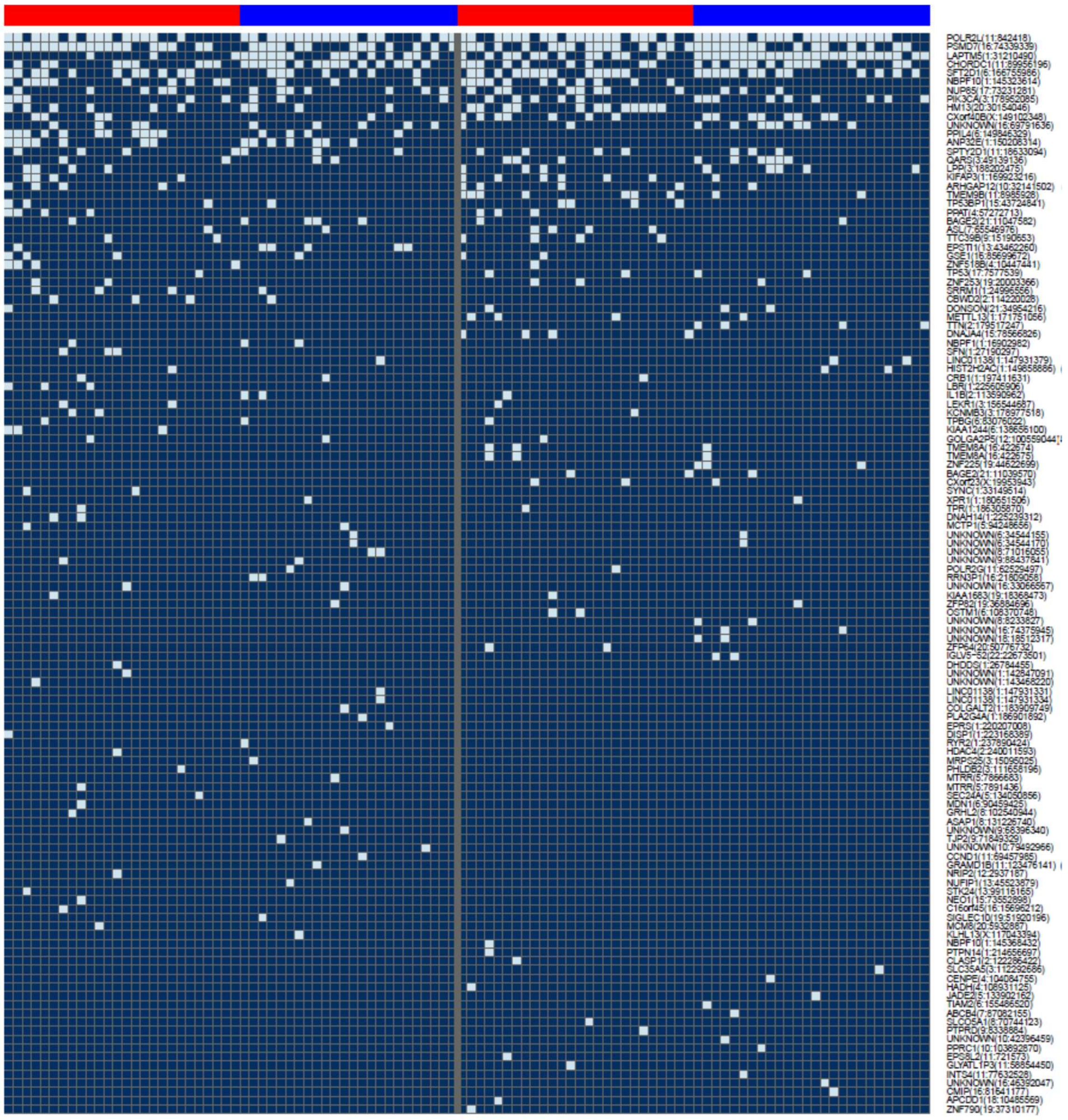

Figure S4: The full list of cell-level mutations of both primary tumor (left) and the lymph node (right) of patient BC07. The annotation is referred to Figure S3.

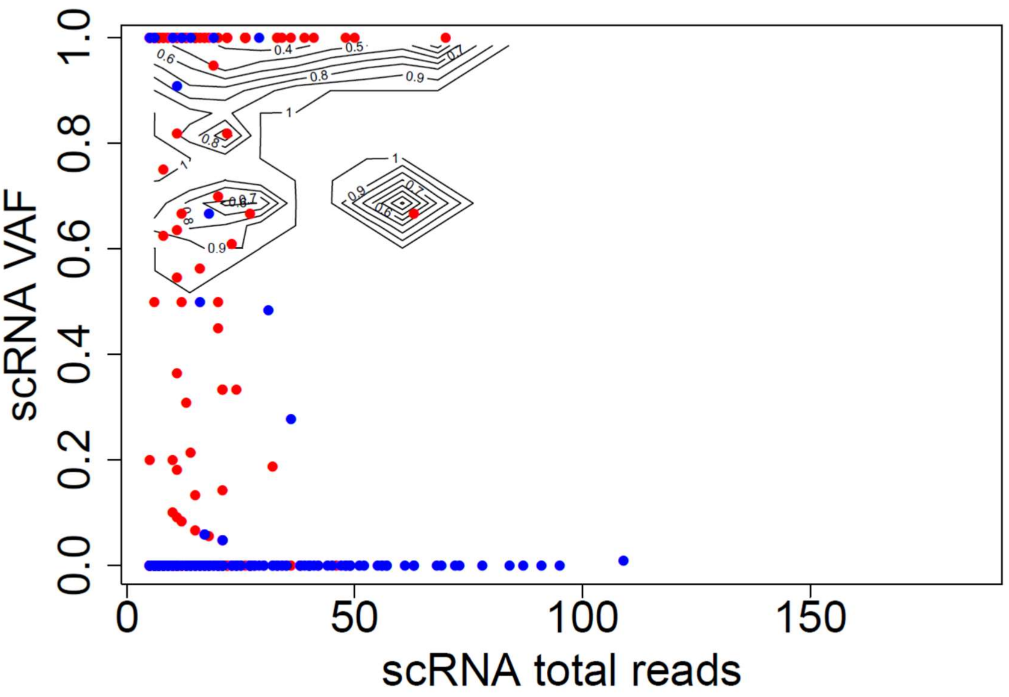

Figure S5. Results of fdr2d from the single cells of primary tumor of patient BC07. The annotation is referred to Figure S1.

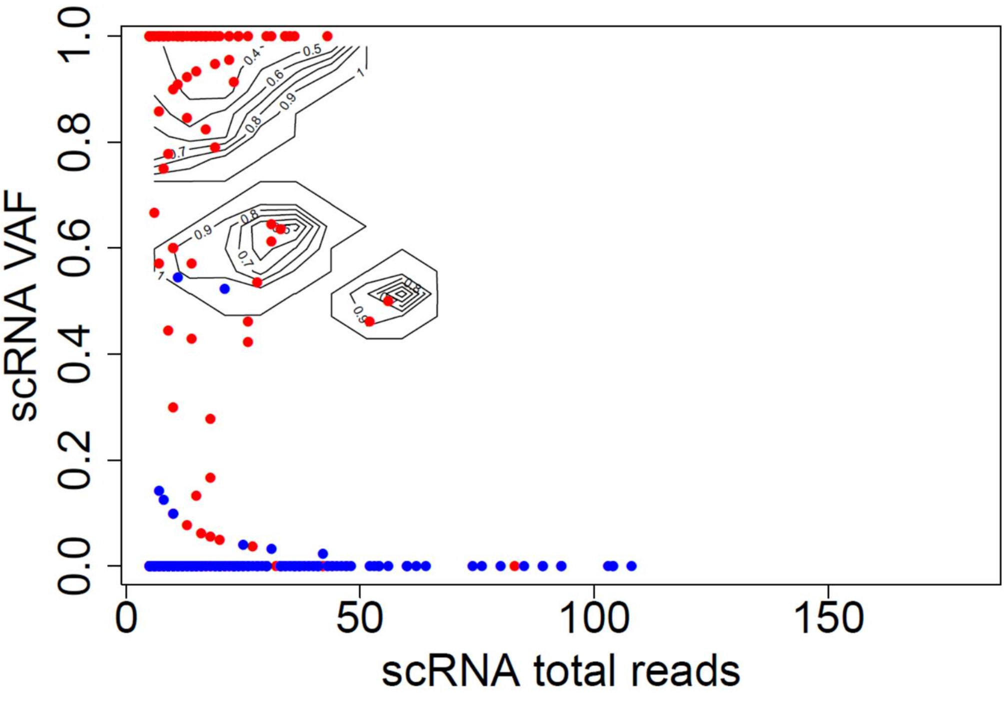

Figure S6. Results of fdr2d from the single cells of lymph node of patient BC07. The annotation is referred to Figure S1.

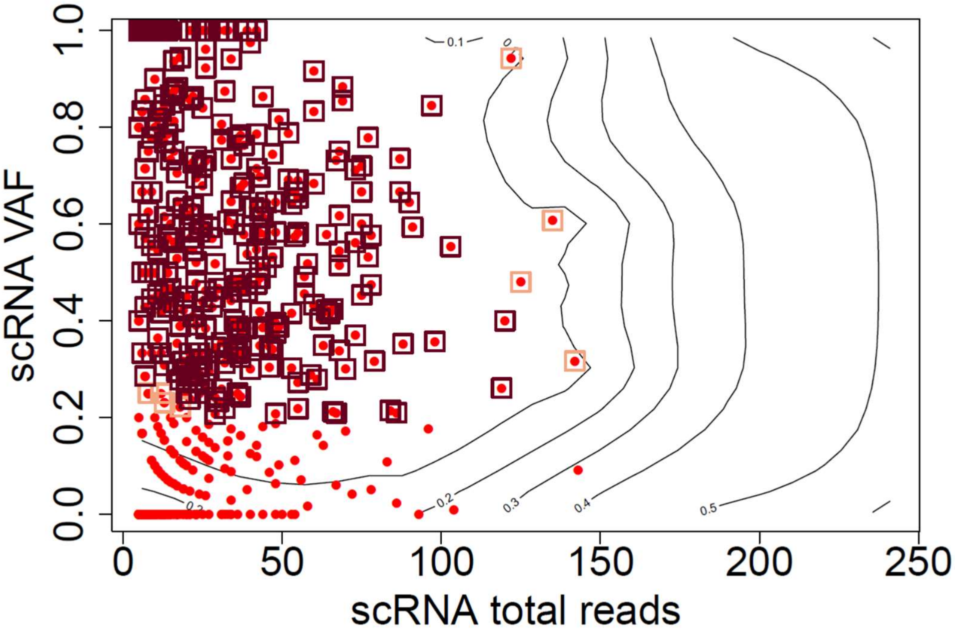

Figure S7. Results of fdr2d from the single cells of control group of MDA-MB-231 cell line. The annotation is referred to Figure S1.

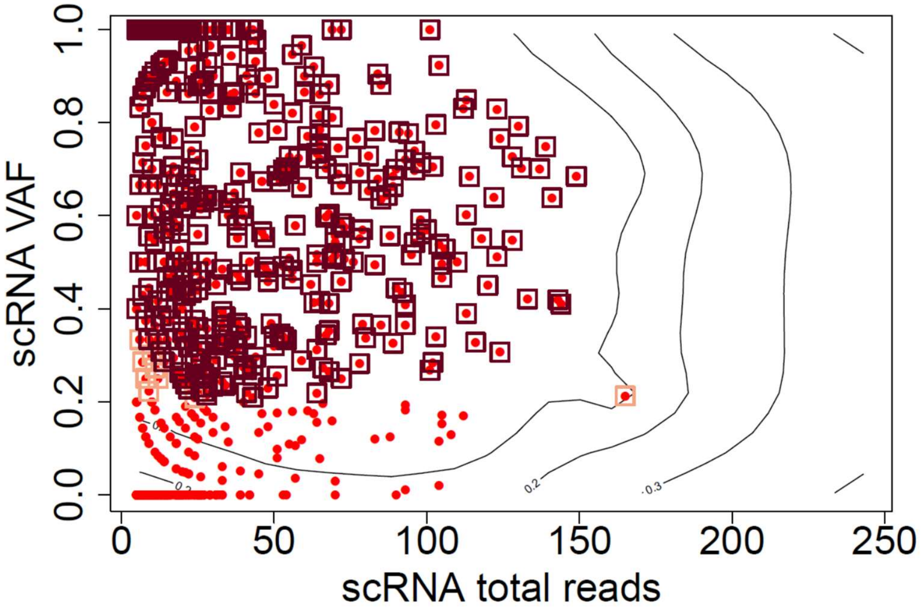

Figure S8. Results of fdr2d from the single cells of treated group of MDA-MB-231 cell line. The annotation is referred to Figure S1.

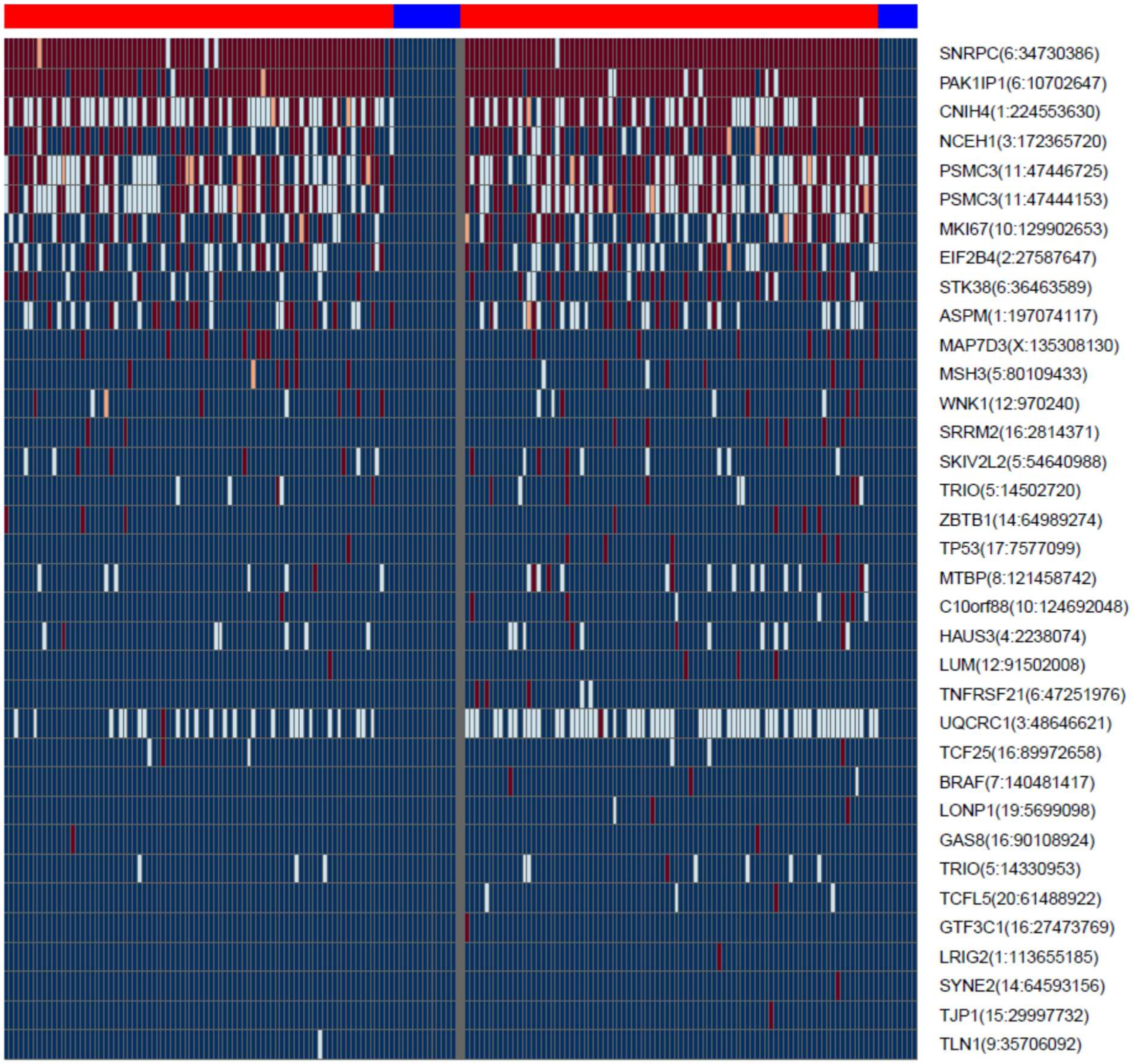

Figure S9: The full list of cell-level mutations of control group (left) and the treated group (right) of MDA-MB-231 dataset. The annotation is referred to Figure S3. The red and blue at the column-side-color band (top) refer the cells and negative controls (no cells).

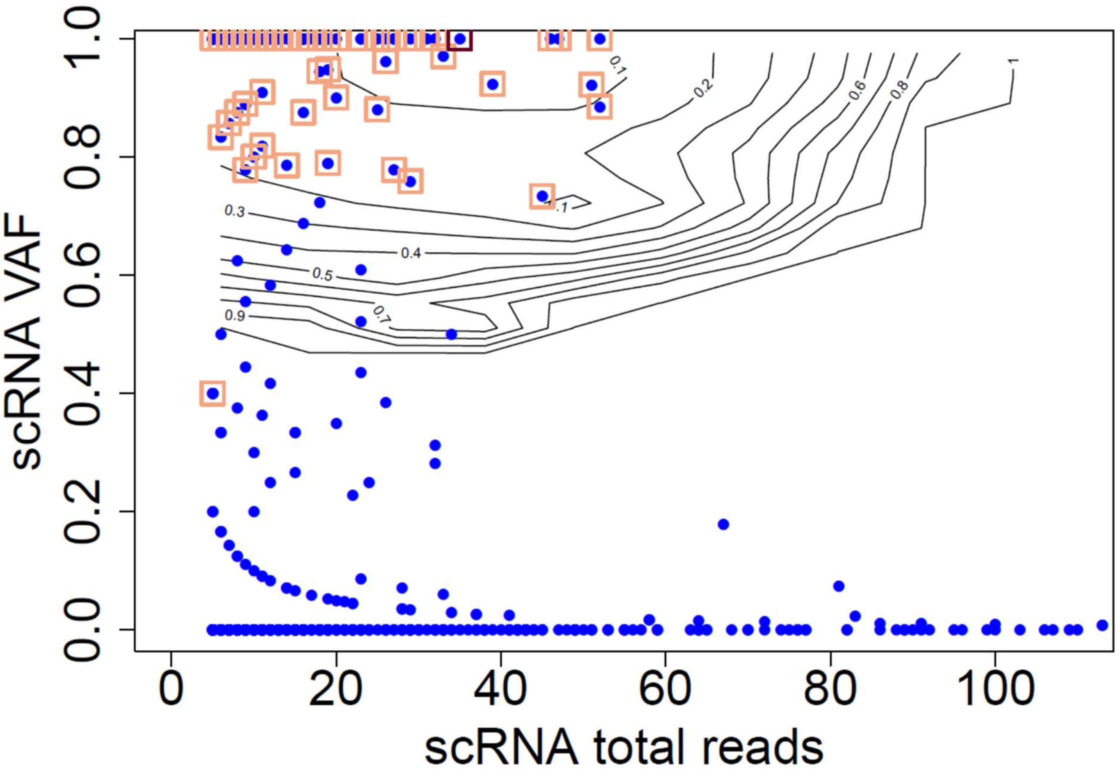

Figure S10: Results of fdr2d from the single cells of the glioblastoma dataset. The annotation is referred to Figure S1.

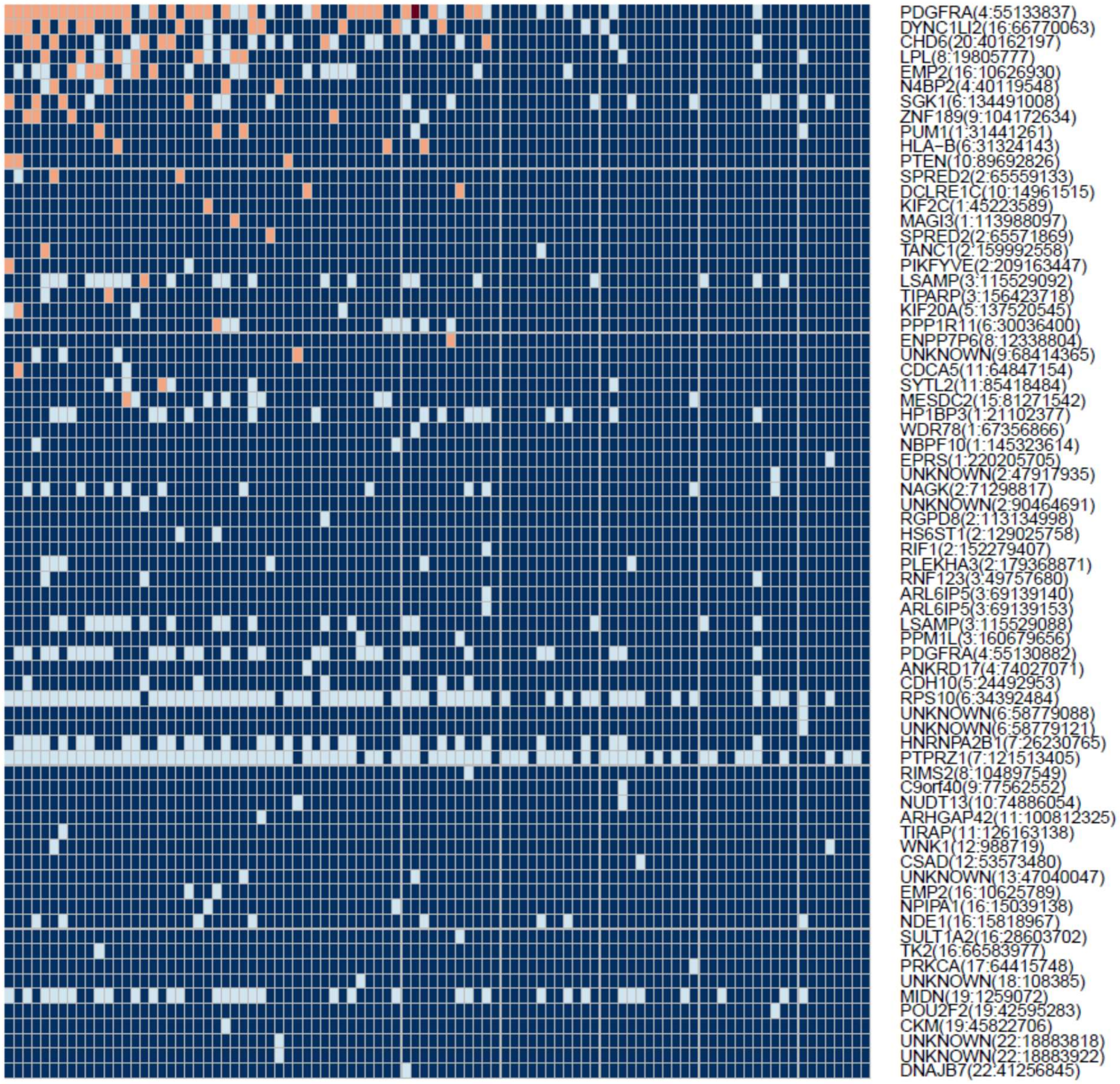

Figure S11: The full list of cell-level mutations of the glioblastoma dataset. The annotation is referred to Figure S3.

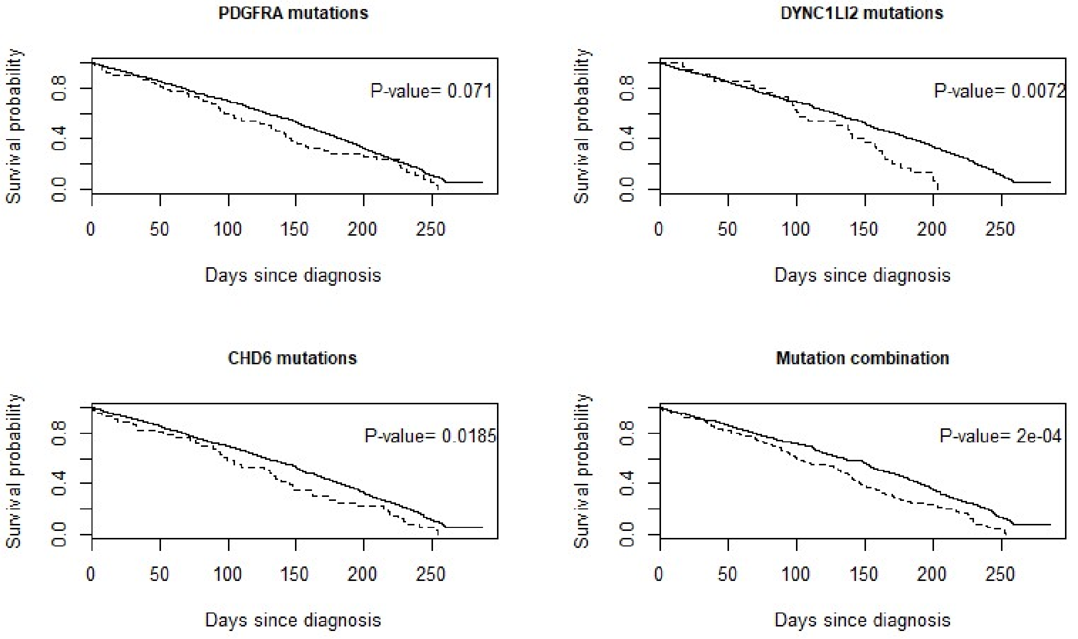

Figure S12: The survival analysis of top three mutational genes discovered by SCmut and their combination using the mutation calls of Mutect from the TCGA-GBM data. The dash line and solid line indicate the patients with and without mutations, respectively.

Detection sensitivity of mutation and coverage threshold

The detection sensitivity depends on statistical power, which in turn depends on the read counts and effect sizes (in this case the underlying mutation rates). So assessing sensitivity from the full matrix of results can be misleading, because a great majority of the matrix entries did not have any reads at all. For example, in BC03 tumour, there were 85 mutation sites from the bulk-cell WES data for which there was any read in at least one of the 33 cells. If we focus on this 85x33 matrix (2805 entries), there were only 412 entries with least one read, and only 238 entries with more than 20 reads. For those 238 entries there were 43 detected mutations (fdr2d < 0.2), so it is not so sparse (see **Figure S13a** below). This detection rate corresponds to what we should expect from the bulk-cell data (**Figure 4e** in the main text).

Sensitivity is of course not always that low. In fact, for the breast cancer cell-line MDA-MB-231 we used in the manuscript, we achieved sensitivity up to 80%, **Figure S13b**, compared to ~40% of the BC03 tumour in **Figure S13a**. The MDA-MB-231 dataset contains a batch of 82 cells from the control group and another batch of 88 cells treated with metformin (treated group). Assuming the drug did not generate new mutations, which seems reasonable from **Figure S13b**, the results from control and treated group can be used as validation of each other. Such validation was shown in **Section 3.4** of the main text.

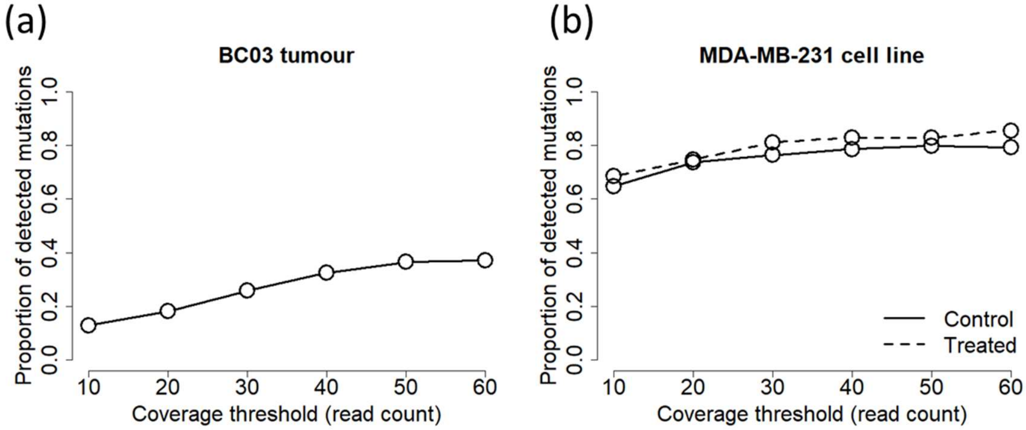

Figure S13. Detection rate of mutation and coverage threshold in a) BC03 tumour and b) the breast cancer cell-line (MDA-MB-231) datasets. Detailed calculations for panel (b): In the control group (n=82 cells), there were 26 confirmed-somatic mutation sites from the COSMIC database (PMID: 27899578) for which there was any read in at least one of the 82 cells. Focusing on this 26x82 matrix (2,132 entries), there were only 414 entries with more than 10 reads, and 268 of those were detected mutations (fdr2d < 0.2). Thus, the proportion of detected mutations is 268/414=65%; for entries more than 60 reads the proportion is ~80%. Similar results were obtained in the treated group.
